# Baxdrostat for uncontrolled and resistant hypertension: rationale and design of the Phase 3 clinical trials BaxHTN, BaxAsia, and Bax24

**DOI:** 10.1038/s41440-025-02297-7

**Published:** 2025-08-25

**Authors:** John M. Flack, Michel Azizi, Jenifer M. Brown, Jamie P. Dwyer, Erika S. W. Jones, Aina S. Lihn, Lylian Liu, Daniel S. Olsson, Shira Perl, Hirotaka Shibata, Ji-Guang Wang, Ulrica Wilderäng, Janet T. Wittes, Bryan Williams

**Affiliations:** 1 Departments of Medicine and Population Science and Policy, Southern Illinois University, Springfield, IL USA; 2 Hypertension Department, Hôpital Européen Georges Pompidou, Paris, France; 3 Department of Medicine, Division of Cardiovascular Medicine, Brigham and Women’s Hospital, Boston, MA USA; 4 Division of Nephrology and Hypertension, University of Utah, Salt Lake City, UT USA; 5 Department of Medicine, Division of Nephrology and Hypertension, Groote Schuur Hospital, University of Cape Town, Cape Town, South Africa; 6 R&D BioPharmaceuticals, Late-Stage Development, Cardiovascular, Renal and Metabolism (CVRM), AstraZeneca, Gothenburg, Sweden; 7 R&D China, Clinical Science, Cardiovascular, Renal, Metabolism (CVRM) and Safety, AstraZeneca, Shanghai, China; 8 R&D BioPharmaceuticals, Late-Stage Development, Cardiovascular, Renal and Metabolism (CVRM), AstraZeneca, Mölndal, Sweden; 9 R&D BioPharmaceuticals, Late-Stage Development, Cardiovascular, Renal and Metabolism (CVRM), AstraZeneca, Gaithersburg, MD USA; 10 Department of Endocrinology, Metabolism, Rheumatology and Nephrology, Faculty of Medicine, Oita University, Oita, Japan; 11 The Shanghai Institute of Hypertension, Ruijin Hospital, Shanghai Jiao Tong University School of Medicine, Shanghai, China; 12 Wittes LLC, Washington, DC USA; 13 Institute of Cardiovascular Science and National Institute for Health Research, UCL Hospitals Biomedical Research Centre, University College London, London, UK

**Keywords:** Aldosterone, Aldosterone synthase inhibitor, Antihypertensive agents, Baxdrostat, Hypertension

## Abstract

Inappropriately elevated aldosterone is a common feature of uncontrolled hypertension (uHTN) and resistant hypertension (rHTN), and is a major pathophysiological driver of adverse cardiorenal outcomes beyond elevated blood pressure (BP). Baxdrostat is a selective aldosterone synthase inhibitor that has demonstrated dose-dependent seated office systolic BP (SBP) lowering in a Phase 2 trial of patients with rHTN. Here, we report the design of the baxdrostat hypertension Phase 3 program. BaxHTN (NCT06034743), BaxAsia (NCT06344104), and Bax24 (NCT06168409) are randomized, multi-national, double-blind, placebo-controlled Phase 3 trials evaluating the efficacy and safety of baxdrostat 1 and/or 2 mg versus placebo. BaxHTN includes patients with uHTN or rHTN, BaxAsia includes patients with uHTN or rHTN primarily from Asia, and Bax24 includes patients with rHTN. Eligibility criteria include age ≥18 years, mean seated office SBP of ≥140 mmHg to <170 mmHg at screening, and ≥2 antihypertensive treatments of different classes for ≥4 weeks before screening. BaxHTN and BaxAsia have four sequential periods following placebo run-in: 12-week double-blind; 12-week open-label; 8-week randomized withdrawal; 20-week open-label. Bax24 has a placebo run-in and 12-week double-blind period. Primary endpoints are changes from baseline to Week 12 in mean seated office SBP (BaxHTN and BaxAsia) and ambulatory 24-h average SBP (Bax24). Safety and tolerability are also assessed. The Baxdrostat hypertension Phase 3 program will assess efficacy, long-term sustained effect, and safety profile in patients with hypertension across multiple geographies. The trials will evaluate the BP lowering efficacy of aldosterone synthase inhibition as a novel treatment for uHTN and rHTN.

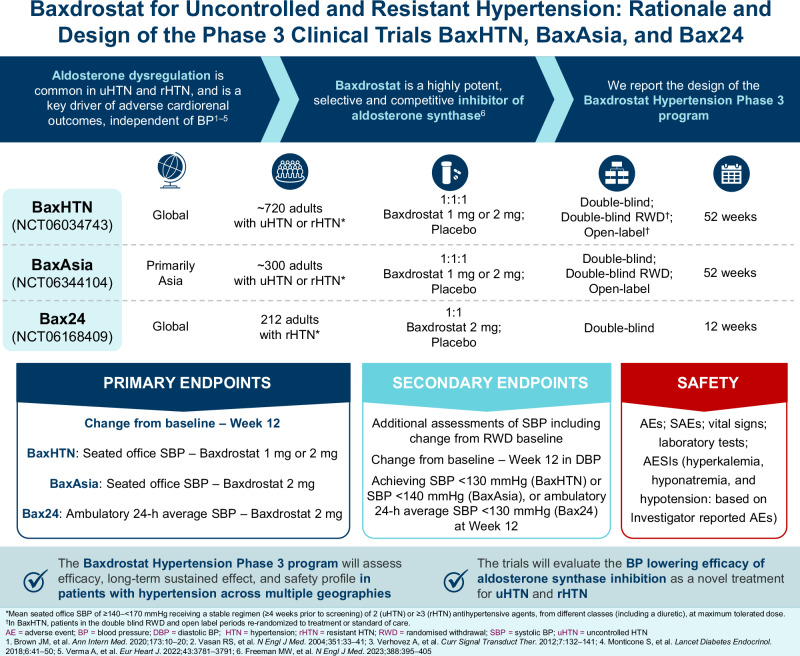

## Introduction

Worldwide, ~1.3 billion adults have hypertension, a leading preventable risk factor for cardiovascular disease and premature mortality [1–4]. Approximately 20% of all deaths are attributed to elevated blood pressure (BP), with a global economic burden of $370 billion, equivalent to around 10% of overall healthcare expenditure [4, 5]. Thus, hypertension is a global health priority that requires effective management to reduce BP-related disease burden.

Hypertension treatment initially involves lifestyle modifications and pharmacological therapies including angiotensin-converting enzyme inhibitors (ACEis), angiotensin II receptor blockers (ARBs), calcium channel blockers (CCBs), and thiazide/thiazide-like diuretics. If BP targets are not achieved within 3–6 months, subsequent addition of mineralocorticoid receptor (MR) antagonists (MRAs), beta blockers, alpha blockers, or central alpha-2 adrenergic receptor agonists are recommended [6–8]. However, ~80% of patients have uncontrolled hypertension (uHTN), with BP levels above target while being treated with <3 antihypertensive therapies [8, 9]. Furthermore, ~15% have resistant hypertension (rHTN), with persistently high BP despite the use of ≥3 antihypertensive therapies at optimal doses, including a diuretic and preferably a renin-angiotensin system (RAS) inhibitor and CCB [6, 8, 10–13].

Patients with uHTN or rHTN have an increased risk of adverse cardiovascular and cerebrovascular events, chronic kidney disease, and death [11, 14–16]. To improve outcomes, it is currently recommended to add a steroidal MRA (such as spironolactone/eplerenone) to the antihypertensive regimen of patients with rHTN [17, 18]. However, spironolactone is not selective towards the MR, and its use is limited by adverse effects including gynecomastia, sexual dysfunction, and menstrual irregularities [19–21], while eplerenone is less potent and not widely available for the treatment of hypertension [8].

A common feature of uHTN and rHTN is aldosterone levels remaining inappropriately elevated despite suppressed renin, angiotensin II, and high sodium intake that increases intravascular volume, or in response to blockade of the RAS [22–25]. The spectrum of aldosterone dysregulation includes not only primary aldosteronism but also borderline or sub-clinical manifestations of elevated aldosterone [23, 26]. MRAs can block the pathophysiological effects of aldosterone, especially at the level of the heart and the kidney; however, they induce a counter-regulatory increase in plasma renin and aldosterone concentrations and stimulate MR-independent effects of aldosterone [27–29]. Moreover, aldosterone exerts genomic and nongenomic effects that MRAs may not fully block [30, 31]. For example, aldosterone has been reported to induce changes in sodium transport, increases in intracellular calcium and pH, and increases in reactive oxygen species that occur too rapidly for genomic effects, and are not inhibited by blocking transcription and translation [31]. Moreover, short-term aldosterone-induced increases in pH and intracellular calcium in cortical collecting duct cells, and increases in nitric oxide synthase activity and sodium–proton exchanger activity in vascular smooth muscle cells, and vasoconstriction of coronary arteries do not appear to be inhibited by MRAs [30, 31]. However, some non-genomic effects of aldosterone, such as vascular smooth muscle cell proliferation, do appear to be mediated through the MR [30]. Studies suggest that aldosterone and its MR may colocalize with, and transactivate, the epidermal growth factor receptor, whereas G-protein coupled receptor 30 (GPR30) has been proposed as an alternative receptor for aldosterone that could be responsible for actions not inhibited by MRAs [31].

Unlike MRAs, aldosterone synthase inhibitors (ASIs) competitively inhibit aldosterone synthase (CYP11B2), a member of the cytochrome P450 family that catalyzes the final three rate-limiting steps in aldosterone synthesis by converting 11-deoxycorticosterone (11-DOC) to aldosterone in the zona glomerulosa of the adrenal cortex [32–34]. By decreasing aldosterone secretion from the adrenal glands and circulating concentrations, ASIs reduce MR activation of aldosterone target organs [32, 35–37]. Baxdrostat, a highly potent ASI is >100 fold more selective for aldosterone synthase (CYP11B2) than 11β-hydroxylase (CYP11B1) in vitro (Supplementary Fig. 1), the enzyme responsible for cortisol synthesis in the zona fasciculata of the adrenal cortex [38]. In healthy individuals on a liberal sodium diet, baxdrostat demonstrated dose-related decreases in plasma and urine aldosterone concentrations, accompanied by dose-related increases in plasma 11-DOC concentrations, thus confirming CYP11B2 inhibition but without any change in plasma cortisol and adrenocorticotropic hormone concentrations [38]. Baxdrostat also has a favorable pharmacokinetic profile in healthy individuals, as it is rapidly absorbed (0.5–2 h) and has a plasma half-life of ~30 h, allowing for once daily (QD) dosing in patients with hypertension [35, 38].

In the Phase 2 BrigHTN trial (NCT04519658), treatment with baxdrostat 1 and 2 mg QD, but not 0.5 mg QD, dose-dependently reduced seated office systolic BP (SBP) in patients with rHTN at Week 12 versus placebo when administered in addition to ≥3 antihypertensive treatments including a diuretic. All doses of baxdrostat reduced plasma and urine aldosterone concentrations, while the two highest doses (1 and 2 mg) increased plasma renin activity. Dose-related decreases in estimated glomerular filtration rate (eGFR) and increases in serum potassium levels were also observed; however, there were no reports of adrenocortical insufficiency, treatment-related serious adverse events (SAEs), severe hyperkalemia, or death [36].

In the Phase 2 HALO trial, treatment with baxdrostat 0.5, 1, or 2 mg QD for 8 weeks in patients with uHTN did not significantly reduce seated office SBP or diastolic BP (DBP) versus placebo; however, all doses decreased serum aldosterone concentrations [39]. The HALO trial failed to demonstrate the BP lowering efficacy of baxdrostat, which may be due to several factors including 1) a large placebo effect (−16.6 mmHg reduction in seated office SBP) possibly because of increased adherence to background therapy, and 2) non-adherence to baxdrostat as suggested by changes in aldosterone excretion and plasma renin levels being less than those observed in BrigHTN and plasma drug concentrations being very low (<0.2 ng/mL) in over a third of patients [40]. A post hoc analysis of patients with baxdrostat plasma concentrations indicative of good adherence suggests that a placebo-corrected reduction in SBP of 7.9 mmHg was achieved when baxdrostat was administered as intended [40]. Given the shortcomings of the HALO trial, better designed Phase 3 trials are needed to assess the efficacy, safety, and tolerability of baxdrostat in a large number of patients with uHTN and rHTN. The baxdrostat Phase 3 clinical trial program in hypertension (BaxHTN [NCT06034743] [41], BaxAsia [NCT06344104] [42], and Bax24 [NCT06168409] [43]) will evaluate the long-term maintenance of baxdrostat’s antihypertensive effect in addition to background antihypertensive therapy in patients across geographies.

## Methods

### Trial designs and objectives

BaxHTN, BaxAsia, and Bax24 are randomized, multi-national, multi-center, double-blind, placebo-controlled Phase 3 trials recruiting patients with uHTN and rHTN (see Supplementary methods for trial locations).

BaxHTN and BaxAsia will compare the difference in seated office SBP change from baseline to Week 12 between baxdrostat 1 and/or 2 mg QD and placebo in patients with uHTN or rHTN who are maintained on their background antihypertensive medications. BaxAsia will include patients primarily from Asia. The 12-week Bax24 study is specifically designed to assess the effects of baxdrostat 2 mg QD on ambulatory 24-hour SBP in patients with rHTN (Supplementary Table 1).

### Eligibility criteria

The three trials have similar inclusion criteria (Table 1); however, Bax24 only includes patients with rHTN. At screening, eligible patients are aged ≥18 years with a mean seated office SBP of ≥140 mmHg to <170 mmHg by automated office BP measurement (AOBPM), receiving a stable regimen of two (uHTN) or ≥3 (rHTN) antihypertensive treatments of different classes at the maximum tolerated dose, including a diuretic, for ≥4 weeks before screening, an eGFR of ≥45 mL/min/1.73 m^2^, and a serum potassium level of ≥3.5 to <5.0 mmol/L. Randomization criteria include a baseline mean seated office SBP of ≥135 mmHg for BaxHTN and BaxAsia, and a mean 24 hour ambulatory SBP of ≥130 mmHg for Bax24.

**Table 1:** Inclusion and exclusion criteria for BaxHTN, BaxAsia, and Bax24

BaxHTN	BaxAsia	Bax24
**Inclusion criteria**		
Age ≥18 years		
Mean seated SBP on AOBPM ≥ 140 mmHg^a^ and <170 mmHg at screening		
uHTN: stable regimen (≥4 weeks prior to screening) of two antihypertensive medications, from different therapeutic classes (≥1 must be a diuretic), at maximum tolerated dose. Beta-blockers used to treat other conditions should not be counted		–
rHTN: stable regimen (≥4 weeks prior to screening) of ≥3 antihypertensive medications, from different therapeutic classes (≥1 must be a diuretic), at maximum tolerated dose. Beta-blockers used to treat other conditions should not be counted		
eGFR ≥45 mL/min/1.73 m^2^		
Serum K^+^ level ≥3.5 and <5.0 mmol/L		
**Exclusion criteria**		
Mean seated SBP on AOBPM ≥ 170 mmHg or DBP of ≥110 mmHg	Mean seated SBP on AOBPM ≥ 170 mmHg or DBP of ≥105 mmHg	Mean seated SBP on AOBPM ≥ 170 mmHg or DBP of ≥110 mmHg
Has a secondary cause of hypertension: renal artery stenosis, uncontrolled or untreated hyperthyroidism, uncontrolled or untreated hypothyroidism, pheochromocytoma, Cushing’s syndrome, aortic coarctation (primary aldosteronism and sleep apnea are permitted)		
NYHA functional HF class IV		
Heart rate <45 or >110 beats/min in a resting position, as per vital signs assessment		
Medical history of stroke, ACS, HE, or hospitalization for HF (within 6 months prior to screening)		
Serum Na^+^ <135 mmol/L		
Current or prior treatment (within 4 weeks prior to screening) with ARBs and ACEis (taken simultaneously)	Current or prior treatment (within 4 weeks prior to screening) with ACEis, ARBs or ARNI (taken simultaneously)	Current or prior treatment (within 4 weeks prior to screening) ARBs and ACEis (taken simultaneously)
Uncontrolled diabetes with HbA1c > 10.0% (86 mmol/mol)		Uncontrolled diabetes with HbA1c > 9.5% (80 mmol/mol)
Treatment with any MRAs, potassium-sparing diuretic, direct renin inhibitor, or antiarrhythmic medications (within 4 weeks prior to screening) or K^+^ binders within 2 months prior to screening. Beta blockers and calcium channel blockers classified as Class II/IV antiarrhythmics used for indications other than arrhythmia, and digoxin are permitted		

*ACEi* angiotensin-converting enzyme inhibitor, *ACS* acute coronary syndrome, *AOBPM* automated office blood pressure measurement, *ARB* angiotensin II receptor blocker, *ARNI* angiotensin receptor/neprilysin inhibitor, *DBP* diastolic blood pressure, *eGFR* estimated glomerular filtration rate, *HbA1c* glycosylated hemoglobin, *HE* hypertensive encephalopathy, *HF* heart failure, *K*^+^ potassium, *MRA* mineralocorticoid receptor antagonist, *Na*^+^ sodium, *NYHA* New York Heart Association, *rHTN* resistant hypertension, *SBP* systolic blood pressure, *uHTN* uncontrolled hypertension

^a^At randomization: baseline mean seated office SBP of ≥135 mmHg for BaxHTN and BaxAsia, and a mean ambulatory SBP of ≥130 mmHg for Bax24

Patients are excluded if they have a mean seated office SBP/DBP of ≥170 mmHg/≥110 mmHg (BaxHTN and Bax24) or ≥170 mmHg/≥105 mmHg (BaxAsia) by AOBPM, prior treatment (within 4 weeks before screening) with MRAs, antiarrhythmic medications, or potassium-sparing diuretics, serum sodium level <135 mmol/L, or uncontrolled diabetes.

### Trial schedule and interventions

**Fig. 1:**
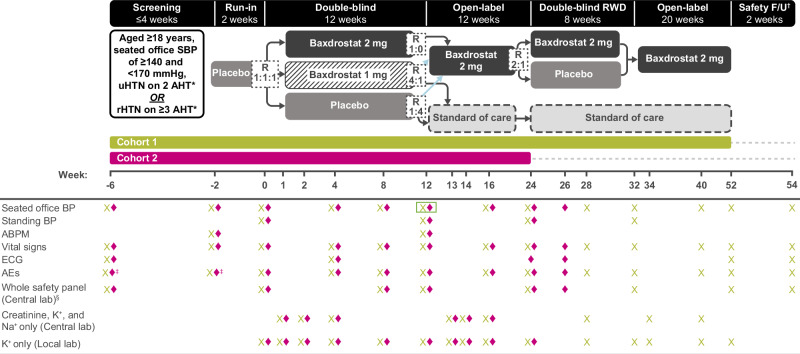
BaxHTN study design. Primary endpoint: change from baseline in seated office SBP at Week 12. *Stable regimen of AHT from different therapeutic classes (including a diuretic), at maximum tolerated dose, for ≥4 weeks prior to screening. Beta blockers used to treat other conditions are not included as AHTs. ^†^For Cohort 2, the 2-week safety follow-up period occurs at Weeks 24–26. ^‡^SAEs only. ^§^The whole safety panel includes whole blood analysis, urinalysis, and serum clinical chemistry. Creatinine and Na^+^ will be measured and collected at every visit that is scheduled between baseline and the end of the study (Central lab). K^+^ will be measured at every visit that is scheduled between baseline and the end of the study (Central lab and Local lab). Urinalysis is only performed at baseline. Urine pregnancy test will be performed at screening and baseline (and ad-hoc if required by local authorities or at the discretion of the investigator). ABPM ambulatory blood pressure monitoring, AE adverse event, AHT anti-hypertensive treatment, BP blood pressure, ECG electrocardiogram, F/U follow-up, K^+^ potassium, Na^+^ sodium, rHTN resistant hypertension, R randomization, RWD randomized withdrawal, SAE serious adverse event, SBP systolic blood pressure, uHTN uncontrolled hypertension

**Fig. 2:**
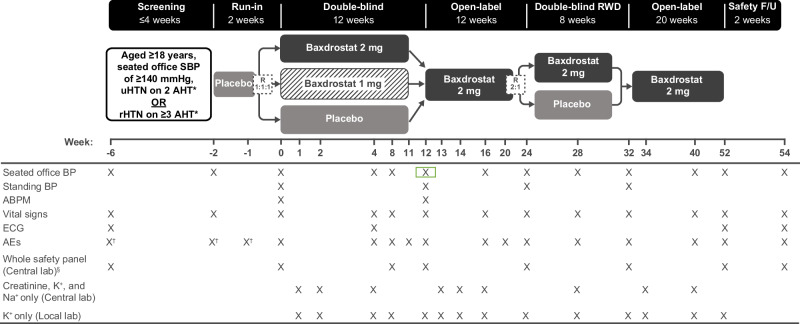
BaxAsia study design. Primary endpoint: change from baseline in seated office SBP at Week 12. *Stable regimen of AHT from different therapeutic classes (including a diuretic), at maximum tolerated dose, for ≥4 weeks prior to screening. Beta blockers used to treat other conditions are not included as AHTs. ^†^SAEs only. ^§^The whole safety panel includes whole blood analysis, urinalysis, and serum clinical chemistry. Creatinine and Na^+^ will be measured at visits scheduled between baseline and the end of the study (Central lab). K^+^ will be measured at visits scheduled between baseline and the end of the study (Central lab and Local lab). Urinalysis is only performed at baseline. Urine pregnancy test will be performed at screening and baseline (and ad-hoc if required by local authorities or at the discretion of the investigator). ABPM ambulatory blood pressure monitoring, AE adverse event, AHT anti-hypertensive treatment, BP blood pressure, ECG electrocardiogram, F/U follow-up, K^+^ potassium, Na^+^ sodium, rHTN resistant hypertension, R randomization, RWD randomized withdrawal, SAE serious adverse event, SBP systolic blood pressure, uHTN uncontrolled hypertension

**Fig. 3:**
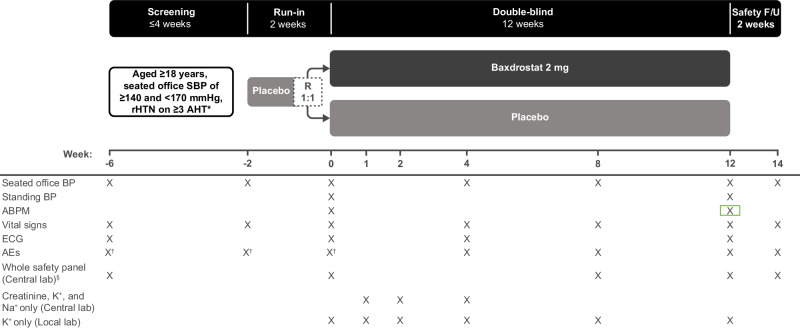
Bax24 study design. Primary endpoint: change from baseline in ambulatory 24-h average SBP. *Stable regimen of AHT from different therapeutic classes (including a diuretic), at maximum tolerated dose, for ≥4 weeks prior to screening. Beta blockers used to treat other conditions are not included as AHTs. ^†^SAEs only. ^§^The whole safety panel includes whole blood analysis, urinalysis, and serum clinical chemistry. Creatinine and Na^+^ will be measured at visits scheduled between baseline and the end of the study (Central lab). K^+^ will be measured at visits scheduled between baseline and the end of the study (Central lab and Local lab). Urinalysis is only performed at randomization. Urine pregnancy test will be performed at screening and randomization (and ad-hoc if required by local authorities or at the discretion of the investigator). ABPM ambulatory blood pressure monitoring, AE adverse event, AHT anti-hypertensive treatment, BP blood pressure, ECG electrocardiogram, F/U follow-up, rHTN resistant hypertension, R randomization, SAE serious adverse event, SBP systolic blood pressure

Figures 1–3 illustrate the trial designs. In all three trials, following a 4-week screening period, eligible patients enter a 2-week single-blind run-in period during which they receive placebo in addition to background antihypertensive treatment (study personnel will witness these being taken) which is maintained throughout the trials. This period is designed to decrease the placebo effect following baseline.

In BaxHTN and BaxAsia, patients who meet eligibility criteria at the end of the run-in period are stratified by uHTN or rHTN status and seated office SBP (<145 or ≥145 mmHg), and randomized 1:1:1 to baxdrostat 1 mg QD, baxdrostat 2 mg QD, or placebo for the 12-week double-blind period (Figs. 1 and 2). In BaxHTN, the first ~450 randomized patients are followed for the full 52 weeks (Cohort 1) and the following ~270 patients are followed for 24 weeks (12-week double-blind period and 12-week open-label period only; Cohort 2). In Bax24, patients who continue to meet eligibility criteria at the end of the run-in period are stratified by mean ambulatory SBP (<140 or ≥140 mmHg) and randomized 1:1 to baxdrostat 2 mg QD or placebo for the 12-week double-blind period (Fig. 3; see Supplementary methods for a description of randomization methods).

After the double-blind period, BaxHTN and BaxAsia have three additional study periods: a 12-week open-label period where patients receive baxdrostat 2 mg QD or standard-of-care (SoC), an 8-week 2:1 randomized withdrawal (RWD) period where patients receive baxdrostat 2 mg QD or placebo, and a further 20-week open-label period where patients receive baxdrostat 2 mg QD (Figs. 1 and 2).

During the 12-week open-label period of BaxHTN, patients randomized to baxdrostat 2 mg QD continue that dose, patients randomized to baxdrostat 1 mg QD are randomized 4:1 to baxdrostat 2 mg and SoC, and patients randomized to placebo are randomized 1:4 to baxdrostat 2 mg and SoC. SoC in BaxHTN refers to the continuation of background antihypertensive treatment with or without additional agents, such as MRAs and potassium-sparing diuretics, at the discretion of the investigator (Fig. 1). During the 12-week open-label period of BaxAsia, patients receive baxdrostat 2 mg QD (Fig. 2).

At the end of the 12-week open-label period, patients from BaxHTN Cohort 1 (Fig. 1) and BaxAsia (Fig. 2) are re-stratified by baseline hypertension (uHTN or rHTN) and seated office SBP (BaxHTN: <130 or ≥130 mmHg; BaxAsia: <140 or ≥140 mmHg) and re-randomized 2:1 to either baxdrostat 2 mg QD or placebo for the 8-week RWD period. After the 8-week RWD period, patients from BaxHTN Cohort 1 (Fig. 1) and BaxAsia (Fig. 2) enter a second open-label period where they receive baxdrostat 2 mg QD for 20 weeks. The open-label periods allow additional long-term exposure data to be gathered. The RWD period is included to assess persistent efficacy of the study treatment, while the second randomization is intended to maintain the blinding of the initial treatment assigned in the double-blind period.

At the end of the treatment periods for each trial, patients enter a 2-week safety follow-up, which allows investigators to continue identifying and appropriately managing any risks associated with baxdrostat.

### Assessments and procedures

Figures 1–3 illustrate the schedule of key assessments for each trial. Three seated office BP measurements are recorded at trough using an adapted cuff on the same arm with the highest BP value throughout the trial (each ~1 min apart, following an initial 5-min rest period) using a centrally provided AOBPM device (Microlife WatchBP Office 2G) or, at screening only, a local BP monitor (prior to baseline), with the mean defined as the average of the last two seated BP measurements. Following measurement of seated SBP, BP is measured once after ~1 min in a standing position using the AOBPM.

For ambulatory BP monitoring (ABPM), the ABPM device (Microlife WatchBP O3) is placed on the non-dominant arm for ≥25 h (excluding the first hour of BP recordings for analysis). The ABPM device records BP every 20 min during the day (6 AM to 9:59 PM) and every 30 min during the night (10 PM to 5:59 AM). ABPM recordings are transferred without treatment allocation to an independent laboratory for prespecified immediate assessment of acceptance criteria and calculation of 24-h average BP.

Direct observed therapy and pill counts are performed to assess adherence of background antihypertensive treatments and study intervention intake. During the direct observed therapy procedure, study personnel will administer the study intervention and background antihypertensive treatments to patients, and witness these medications being taken by the patients. Patients will then be monitored for ≥1 h for adverse effects.

Vital signs include BP, pulse rate, and body weight measurements. Safety blood analysis (including whole blood count, glycosylated hemoglobin [HbA1c], and serum creatinine, sodium, and potassium), urinalysis variables (blood, protein, and leukocytes) and electrocardiogram (ECG) are monitored at regular intervals. eGFR is monitored systemically throughout the trials by a central laboratory and calculated using creatinine results obtained from blood samples using the 2021 Chronic Kidney Disease Epidemiology Collaboration (CKD-EPI) equation. Local laboratory sample values are used for safety decision-making and central laboratory samples are used for analysis purposes. When assessing hyperkalemia, values from potassium samples analyzed in local laboratories take priority over the results obtained at a central laboratory.

The investigators will assess adverse events (AEs) and SAEs, which include hospitalization and death. AEs of special interest include hyperkalemia (serum potassium >5.0 mmol/L), hyponatremia (serum sodium <135 mmol/L), and hypotension which requires medical intervention. Major adverse cardiovascular events (MACE), defined as cardiovascular death, non-fatal myocardial infarction, non-fatal stroke, and hospitalization for heart failure (MACE-plus) are adjudicated by a blinded Event Adjudication Committee.

### Endpoints

In BaxHTN and BaxAsia, the primary endpoints are change from baseline to Week 12 in seated office SBP by AOBPM. In Bax24, the primary endpoint is change from baseline to Week 12 in ambulatory 24-h average SBP. Secondary and safety endpoints for the three trials are listed in Table 2.

**Table 2:** Endpoints for BaxHTN, BaxAsia, and Bax24

	BaxHTN	BaxAsia	Bax24
**Primary endpoints**	Change from baseline in seated office SBP at Week 12 with baxdrostat 2 mg		Change from baseline in ambulatory 24-h average SBP at Week 12 with baxdrostat 2 mg
	Change from baseline in seated office SBP at Week 12 with baxdrostat 1 mg	–	–
**Secondary endpoints**	Change from RWD baseline (Week 24) in seated office SBP at Week 32 with baxdrostat 2 mg	Change from RWD baseline (Week 24) in seated office SBP at Week 32 with baxdrostat 2 mg	Change from baseline in ambulatory night-time/daytime average SBP at Week 12 with baxdrostat 2 mg
	Change from baseline in seated office SBP at Week 12 with baxdrostat 1 mg or 2 mg, in patients with rHTN	Change from baseline in seated office SBP at Week 12 with baxdrostat 1 mg	Change from baseline in seated office SBP/DBP at Week 12 with baxdrostat 2 mg
	Change from baseline in seated office DBP at Week 12 with baxdrostat 1 mg or 2 mg	Change from baseline in seated office DBP at Week 12 with baxdrostat 1 mg or 2 mg	Achieving ambulatory 24-h average SBP of <130 mmHg at Week 12 with baxdrostat 2 mg
	Achieving seated office SBP <130 mmHg at Week 12 with baxdrostat 1 mg or 2 mg	Achieving seated office SBP <140 mmHg at Week 12 with baxdrostat 1 mg or 2 mg	Change from baseline in ambulatory night-time/daytime/24-h average DBP at Week 12 with baxdrostat 2 mg
	–	Change from baseline in the mean ambulatory 24-hour average SBP at Week 12 with baxdrostat 1 mg or 2 mg, by ABPM	Achieving nocturnal SBP dipping of ≥10% at Week 12 with baxdrostat 2 mg
	–	Change from baseline in seated SBP at Week 12 with baxdrostat 1 mg or 2 mg, in patients with rHTN	–
**Safety endpoints**	AEs, SAEs, and DAEs		
	Vital signs (BP, pulse rate, and body weight)		
	Safety investigations and laboratory tests (ECG, clinical chemistry, hematology)		
	AESIs (hyperkalemia, hyponatremia, and hypotension: based on Investigator reported AEs)		
	MACE (cardiovascular death, non-fatal myocardial infarction, and non-fatal stroke)		–
	MACE-plus (cardiovascular death, non-fatal myocardial infarction, and non-fatal stroke and hospitalization for heart failure)		–

*ABPM* ambulatory blood pressure monitoring, *AE* adverse event, *AESI* adverse event of special interest, *BP* blood pressure, *DAE* discontinuation due to adverse event, *DBP* diastolic blood pressure, *ECG* electrocardiogram, *MACE* major adverse cardiovascular event, *rHTN* resistant hypertension, *RWD* randomized withdrawal, *SAE* serious adverse event, *SBP* systolic blood pressure

All endpoints are versus placebo. Nocturnal dip is defined as the difference between ambulatory daytime average SBP and ambulatory night-time average SBP, expressed as a percentage of the daytime average SBP. Patients who have a ≥10% reduction in night-time SBP compared to daytime SBP are characterized as dippers

### Statistical analysis of primary endpoints

BaxHTN and BaxAsia will enroll ~720 and 300 patients, respectively. These sample sizes provide 98% power (two-sample t-test with a two-sided significance level of 0.025 for baxdrostat versus placebo) for BaxHTN, and 80% power (two-sample t-test with a two-sided significance level of 0.05 for baxdrostat versus placebo) for BaxAsia, to detect a difference of 6 mmHg (assuming an SD of 15 mmHg) in mean changes from baseline to Week 12 in seated office SBP between baxdrostat 1 mg or 2 mg and placebo.

For Bax24, a sample size of approximately 212 patients provides 88% power (two-sample t-test with a two-sided significance level of 0.05 for baxdrostat versus placebo) to detect a difference of 6 mmHg (assuming a common SD of 12 mmHg) in change from baseline to Week 12 in 24-h average ambulatory SBP between baxdrostat 2 mg and placebo.

In all three trials, the primary endpoints are analyzed using an analysis of covariance (ANCOVA) model applied to the intention-to-treat (ITT) population. For BaxHTN and BaxAsia, treatment and hypertension (uHTN or rHTN) are fixed factors, and baseline seated office SBP is a covariate. In Bax24, treatment is a fixed effect and baseline ambulatory 24-h average SBP is included as a covariate.

Multiple imputation is conducted for patients in BaxHTN and BaxAsia with missing data, in line with the primary estimands. Missing data following treatment discontinuation are imputed with a retrieved dropouts method based on a missing not at random (MNAR) assumption. Missing data following initiation of rescue treatment are imputed with the reference-based imputation method multiple imputation washout (MI-WO). Imputation of missing endpoints are based on observed endpoint measurements from patients in the placebo arm assuming the data pattern follows MNAR (for patients in the baxdrostat arm) or missing at random (MAR; for patients in the placebo group). For primary analysis of the primary endpoint in Bax24, missing data will not be imputed. ABPM data will only be imputed for a sensitivity analysis and supplementary analysis.

The ANCOVA model provides least squares (LS) means, LS means differences, standard errors and 95% confidence intervals. Statistical analyses are conducted using SAS® software (SAS Institute Inc., Cary, NC, USA). See Supplementary methods for details on the analysis sets and statistical analyses for the secondary endpoints.

Multiplicity adjustments are used to control the Type I error rate at 0.05 (two-sided) for the primary and secondary efficacy endpoints.

No interim analyses for efficacy are planned. Descriptive statistics will be used for safety data. AEs will be coded using the most recent version of the Medical Dictionary for Regulatory Activities (MedDRA). The MedDRA version used at the start of each study was: BaxHTN and BaxAsia, v26.1; BaxA24, v27.0.

### Ethics and trial oversight

Appropriate Institutional Review Boards/Independent Ethics Committees reviewed and approved the protocols. All patients will provide written informed consent and trials are performed in accordance with ethical principles derived from the Declaration of Helsinki and that are consistent with International Council for Harmonization/Good Clinical Practice, applicable laws and regulations in each participating country, and the AstraZeneca Global Standard of Bioethics.

Data Monitoring Committees will monitor the safety and scientific integrity of each trial. In BaxHTN and BaxAsia, a blinded Event Adjudication Committee will adjudicate all potential MACE and MACE-plus in accordance with adjudication criteria in the Event Adjudication Committee charter.

## Discussion

Inhibition of aldosterone synthase is a novel approach that targets aldosterone dysregulation by inhibiting its production in the adrenal cortex [35, 36, 38].

The primary mineralocorticoid hormone, aldosterone, is implicated in BP regulation since it binds to MRs, which trigger genomic effects in the distal nephron of the kidneys that promote sodium and water retention and potassium loss [23, 44, 45]. Aldosterone dysregulation occurs when aldosterone secretion is no longer adequately suppressed by RAS blockade, sodium loading, or extracellular volume expansion [23]. The condition includes not only primary aldosteronism but also borderline aldosteronism or MR associated hypertension [46, 47]. Increased serum aldosterone is directly related to increased BP and new onset hypertension risk, particularly in primary, or renin-independent, aldosteronism, a prevalent cause of uHTN and rHTN [23, 48–55].

Pathological outcomes of aldosterone dysregulation manifest across a continuous spectrum of severity (Supplementary Fig. 2) [48]. Independent of its renal effects, aldosterone, together with a high-sodium environment, stimulates inflammatory reactions, cellular hypertrophy, matrix formation, and apoptosis in the vessels, heart, and kidneys, promoting organ damage and driving disease progression [56]. A meta-analysis of 31 observational studies showed that, compared to patients with essential hypertension, those with primary aldosteronism were at increased risk of stroke, coronary artery disease, atrial fibrillation, and heart failure [57].

Baxdrostat, a highly selective ASI, targets aldosterone dysregulation through direct inhibition of adrenal aldosterone synthase production. It neutralizes the effects of excess aldosterone and lowers BP, avoiding the limitations of MRAs [35, 36].

BaxHTN and BaxAsia are designed primarily to investigate the effect of baxdrostat on seated office SBP, an outcome of clinical relevance [41, 42]. Importantly, MACE are significantly decreased in patients with hypertension when office SBP is reduced to <140 mmHg [58]. Office SBP targets following treatment have been lowered to <130 mmHg (if tolerated) in all guidelines, based on results of randomized trials showing the cardiovascular benefits of such a strategy [6–8, 10, 59, 60]. Moreover, a meta-analysis of individual patient-level data from 48 randomized trials demonstrated that a 5 mmHg reduction of SBP reduced the risk of MACE by ~10% [61].

Bax24 will complement BaxHTN and BaxAsia, by assessing ambulatory 24-h average SBP [43]. Longitudinal studies conducted in both the general population and patients with hypertension have demonstrated that ambulatory BP provides a more accurate prediction of hypertension outcomes than seated office BP [16, 62–66]. In patients with controlled seated office BP but elevated ambulatory BP, an increased risk of organ damage and adverse cardiovascular events was observed versus patients with controlled ambulatory BP [16]. Assessing 24-h average SBP and night-time average SBP is also important as BP variability and high nocturnal BP levels are associated with increased cardiovascular risk [66]. This suggests seated office BP and ambulatory BP measurements may be complementary in determining hypertension prognosis.

Historically, hypertension trials for MRAs have focused on patients with rHTN [18, 21]. BaxHTN and BaxAsia broaden the population of patients included to those with uHTN despite ongoing background treatment with two antihypertensive treatments, in addition to those with rHTN. This will highlight the potential beneficial impact of treating uHTN before it becomes resistant and therefore increase the external applicability of their results to a broader population of patients of diverse ethnicities [41, 42]. Bax24 will only enroll patients with rHTN using the highest baxdrostat dose (2 mg) as they are more likely to have aldosterone dysregulation than those with uHTN only [43, 67]. Moreover, patients with rHTN have demonstrated favorable BP responses to MRAs and may therefore derive the largest ambulatory BP decreases with baxdrostat [18, 29, 67, 68].

BaxHTN, BaxAsia, and Bax24 have been designed to assess the effect of baxdrostat in addition to standard background hypertension therapy, thus maximizing the relevance of their results to clinical practice [41–43]. Furthermore, placebo use is limited to avoid the risks of uncontrolled BP, and patients are permitted to continue their background antihypertensive treatments. To provide a clear understanding of the long-term safety profile of baxdrostat, the study durations extend to 52 weeks with hyperkalemia (potassium level >5.0 mmol/L), hyponatremia (sodium level <135 mmol/L), and hypotension events that require medical intervention included as AEs of special interest.

By recruiting patients from a wide range of countries, this will potentially further our understanding of ASI use in patients of different ethnicities. This includes patients living in Asia who may have a significantly greater risk of aldosterone dysregulation and hypertension-associated adverse cardiovascular events due to higher dietary salt intake and greater salt sensitivity, compared with patients living in countries outside of Asia such as Australia and New Zealand [69–72].

As with any randomized controlled trial, BaxHTN, BaxAsia, and Bax24 include stringent eligibility criteria that may limit generalizability to certain groups of patients (e.g., those with a history of uncontrolled diabetes, recent adverse cardiovascular events, and prior treatment with certain medications). While it is likely that the unselected hypertension population of the three trials are enriched for disorders of aldosterone dysregulation, including undiagnosed primary aldosteronism, this is not a prerequisite for eligibility. Future studies should aim to assess the prespecified trial endpoints in subpopulations of patients with known aldosterone dysregulation to understand further the role this plays in hypertension outcomes. Furthermore, none of the three trials included cardiovascular and renal outcomes as endpoints; they are designed to focus on BP control; however, cardiac safety will be monitored in all three trials, in addition to eGFR as a prognostic marker of kidney disease. Cardiovascular and renal outcomes trials with baxdrostat are currently ongoing, including two phase 3 trials comparing baxdrostat and dapagliflozin with dapagliflozin alone in patients with chronic kidney disease and hypertension (NCT06742723; NCT06268873) [73, 74]. Another phase 3 trial will compare baxdrostat and dapagliflozin versus baxdrostat alone for reducing risk of heart failure or cardiovascular death in patients with type 2 diabetes and cardiovascular disease (NCT06677060) [75].

The baxdrostat hypertension Phase 3 clinical trial program (BaxHTN, BaxAsia, Bax24) is designed to assess efficacy (via a standard Week 12 endpoint to assess BP) and potential long-term sustained effect (via the RWD component) of baxdrostat in hypertension populations across multiple geographies. The safety profile of baxdrostat will also be examined. These trials will establish the effectiveness of treating elevated BP with aldosterone synthase inhibition, and in turn, may provide a novel therapeutic approach for uHTN and rHTN.

## Supplementary information

supplementary materials
