# Supplementary material for: Baxdrostat for uncontrolled and resistant hypertension: rationale and design of the Phase 3 clinical trials BaxHTN, BaxAsia, and Bax24
Source: Hypertens Res. 2025 Aug 25;48(11):2911–23. doi: 10.1038/s41440-025-02297-7 (PMC12586160; doi:10.1038/s41440-025-02297-7)
Supplement: Supplementary file 1 — supplementary materials [file 41440_2025_2297_MOESM1_ESM.docx]

# **Supplementary Materials**

**Supplementary methods**

**Trial locations**

BaxHTN is being carried out at 263 sites across 29 countries (Argentina, Australia, Austria, Belgium, Bulgaria, Canada, Czechia, Denmark, France, Germany, Hungary, India, Israel, Italy, Japan, Malaysia, Netherlands, Poland, Republic of Korea, Slovakia, South Africa, Spain, Sweden, Taiwan, Thailand, Turkey, United Kingdom [UK], United States [US], Vietnam) between November 2023 and October 2025.

BaxAsia is being carried out at 112 sites across 11 countries (Argentina, Australia, China, Hong Kong, India, Japan, Republic of Korea, Philippines, Russian Federation, Turkey, Vietnam) between April 2024 and May 2026.

Bax24 is being carried out at 119 sites across 22 countries (Argentina, Australia, Belgium, Bulgaria, Canada, Czechia, Germany, Greece, Hungary, Malaysia, Philippines, Poland, Saudi Arabia, Slovakia, South Africa, Spain, Taiwan, Thailand, Turkey, UK, US, Vietnam) between March 2024 and September 2025.

**Analysis sets**

The full analysis set (FAS) is defined as all randomized patients who received ≥1 dose of study treatment. The FAS is used for all efficacy endpoints except those defined in the randomized withdrawal period (RWD). The RWD analysis set is defined as all randomized patients in the RWD period who received ≥1 dose of study treatment in the RWD period. The RWD analysis set is used for all efficacy endpoints in the RWD period. The ambulatory blood pressure monitoring (ABPM) analysis set is defined as all patients in the FAS with a completed ABPM session at both run-in and Week 12. The ABPM analysis set is used for all efficacy endpoints regarding ABPM. The safety analysis set (SAS) is defined as all randomized patients who received ≥1 dose of study treatment. The SAS is used for all safety endpoints.

**Randomization**

All patients who meet the eligibility criteria are randomized using an automated Interactive Response Technology/Randomisation and Trial Supply Management (IRT/RTSM) system. Randomization is performed in balanced blocks of fixed size. In BaxHTN, the randomization codes are computer generated and loaded into the interactive voice/web response system (IxRS) database. In BaxAsia and Bax24, the randomization codes are computer generated and loaded into the IRT/RTSM database. Randomization is performed by the investigator via the IRT/RTSM system. No randomization codes are reused. In BaxHTN and BaxAsia, the number of randomized patients in each hypertension subpopulation (uncontrolled hypertension [uHTN]/resistant hypertension [rHTN]) will be monitored to ensure a minimum of ~40% of patients are enrolled in the uHTN/rHTN subpopulations.

**Statistical analysis of secondary endpoints**

In BaxHTN and BaxAsia, change from RWD baseline (Week 24) to Week 32 in seated office systolic blood pressure (SBP) are analyzed using an analysis of covariance (ANCOVA) model with treatment and hypertension at baseline (uHTN or rHTN) as fixed factors, and RWD baseline (Week 24) seated office SBP as a covariate. Change from baseline to Week 12 in seated office SBP in the rHTN population is analyzed using an ANCOVA model with treatment as a factor and baseline seated office SBP value as a covariate. Change from baseline to Week 12 in seated office diastolic BP (DBP) is analyzed in the same way as the analyses of the primary endpoints. Achievement of a seated office SBP of <130 mmHg (BaxHTN) or <140 mmHg (BaxAsia) at Week 12 is analyzed using a logistic regression model including baseline seated office SBP as a covariate and hypertension at baseline (uHTN or rHTN) as a factor. In BaxAsia, change from baseline in the ambulatory 24-hour average SBP measured by ABPM is analyzed using an ANCOVA model with treatment and hypertension at baseline (uHTN or rHTN) as factors, and baseline 24-hour average SBP as a covariate.

In Bax24, the continuous secondary endpoints are analyzed in the same way as the analyses of the primary endpoint. These endpoints include change from baseline to Week 12 in ambulatory night-time average SBP/DBP, change from baseline to Week 12 in ambulatory daytime average SBP/DBP, change from baseline to Week 12 in seated office SBP/DBP, and change from baseline to Week 12 in ambulatory 24-hour average DBP. Achievement of ambulatory 24-hour average SBP <130 mmHg at Week 12 is analyzed using a logistic regression model, including baseline ambulatory 24-hour average SBP as a covariate and treatment as a factor. Achievement of a nocturnal SBP dipping of ≥10% at Week 12 is analyzed using a logistic regression model, including baseline dipping status as a covariate and treatment as a factor.

**Supplemental Tables**

**Supplemental Table 1** Key Similarities and Differences Between BaxHTN, BaxAsia, and Bax24

|  | **BaxHTN** | **BaxAsia** | **Bax24** |
| --- | --- | --- | --- |
| **Type of trial** | Phase 3 | Phase 3 | Phase 3 |
| **Location** | Global | Primarily Asia | Global |
| **Objective** | Evaluate the safety, tolerability, and effect of baxdrostat on reduction of SBP | Evaluate the safety, tolerability, and effect of baxdrostat on reduction of SBP | Evaluate the safety, tolerability, and effect of baxdrostat on reduction of ambulatory SBP |
| **Population** | Adults with  uHTN or rHTN | Adults with  uHTN or rHTN | Adults with  rHTN |
| **Planned total  sample size** | 720 | 300 | 212 |
| **Treatment arms** | Baxdrostat 1 mg  Baxdrostat 2 mg  Placebo  Standard-of-care | Baxdrostat 1 mg  Baxdrostat 2 mg  Placebo | Baxdrostat 2 mg  Placebo |
| **Treatment periods** | Double-blind  Double-blind RWD  Open-label | Double-blind  Double-blind RWD  Open-label | Double-blind |
| **Treatment duration** | 52 weeks | 52 weeks | 12 weeks |
| **Primary endpoint** | Change from baseline in seated office SBP at Week 12 with baxdrostat  1 mg or 2 mg | Change from baseline in seated office SBP at Week 12 with baxdrostat 2 mg | Change from baseline in ambulatory 24-hour average SBP at  Week 12 with baxdrostat 2 mg |

rHTN, resistant hypertension; RWD, randomized withdrawal, SBP, systolic blood pressure; uHTN, uncontrolled HTN.

**Supplemental Fig. 1** Mechanism of action of baxdrostat


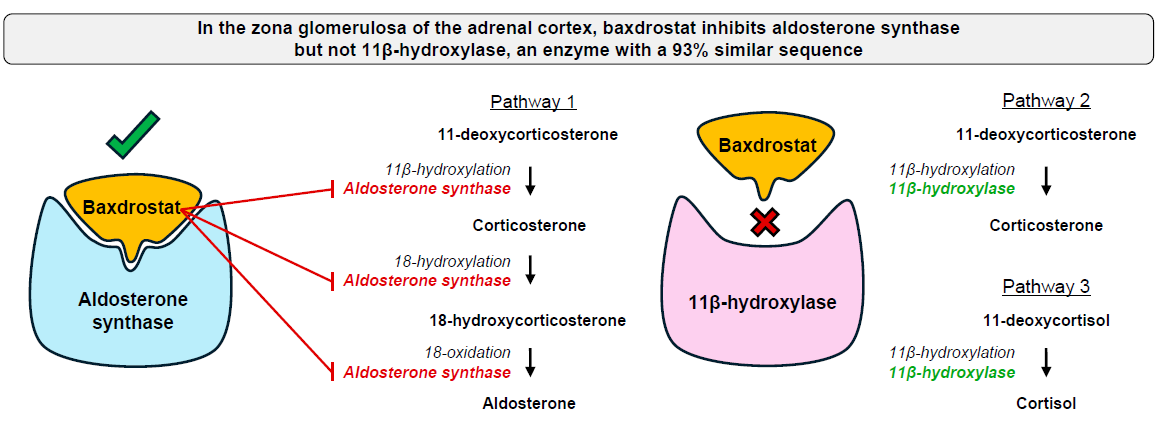


Supplemental Fig. 1 has been adapted with permission from the article Taming resistant hypertension: The promise of novel pharmacologic approaches and renal denervation. Azzam O, et al. Br J Pharmacol. 2023;181:319–339, Copyright © 2023 British Pharmacological Society, published by John Wiley & Sons Ltd.

**Supplemental Fig. 2** Relationship between aldosterone dysregulation and hypertension severity


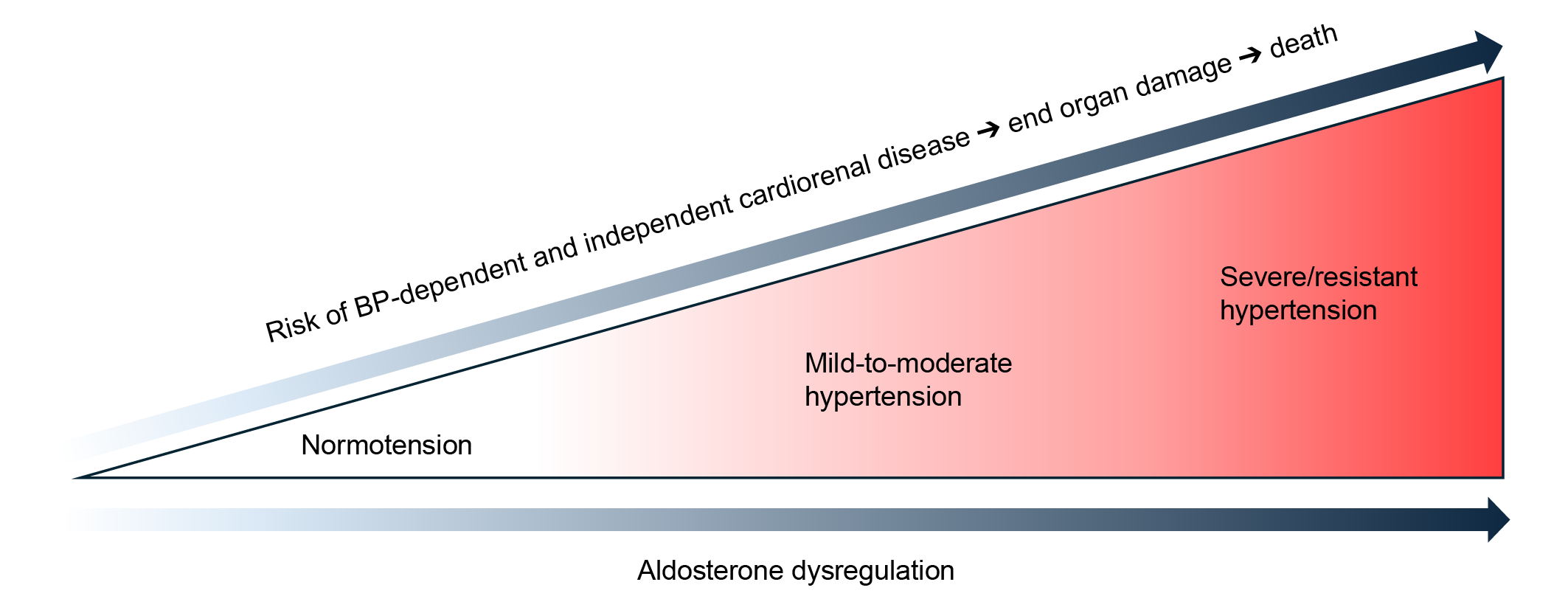


Aldosterone dysregulation occurs as a gradual, continuous process; elevated aldosterone levels are associated with hypertension severity and risk of both BP-dependent and BP-independent adverse cardiorenal disease outcomes. Adverse outcomes resulting from aldosterone dysregulation are manifested across a continuum of severity; however, elevated aldosterone may place patients at risk for cardiovascular disease even before the development of hypertension.

BP, blood pressure.

Supplemental Fig. 2 has been adapted from *Annals of Internal Medicine*, Brown JM, et al. The spectrum of subclinical primary aldosteronism and incident hypertension: a cohort study. Ann Intern Med. 2017:167(9):630–641. Copyright © 2017 American College of Physicians. All Rights Reserved. Reprinted with the permission of the American College of Physicians, Inc.
